# Feasibility and safety of laparoscopic small bowel resections with intracorporal anastomosis in emergency settings – a retrospective single centre study

**DOI:** 10.1007/s00423-026-04188-y

**Published:** 2026-08-11

**Authors:** Jasmin Monika Martina Schmitt, Christoph-Thomas Germer, Sven Flemming, Johan Friso Lock, Florian Seyfried

**Affiliations:** Department of General, Visceral, Transplant, Vascular and Pediatric Surgery, University Hospital of Wuerzburg, Oberdürrbacher Straße 6, 97080 Wuerzburg, Germany

**Keywords:** Minimally invasive surgery, Open surgery, Primary anastomosis, Small bowel resection, EMAC (emergency and acute care surgery)

## Abstract

**Introduction:**

Minimally invasive surgery (MIS) is increasingly applied in complex abdominal surgery and has been associated with potential benefits compared with the open approach (OS). However, evidence on the feasibility and safety on MIS small bowel resections (SBR) in the emergency setting remains limited.

**Methods:**

The study included patients requiring emergency SBR with primary anastomosis between 01/2020 and 04/2025; data were collected from the hospital information system at a tertiary referral centre. Patient outcomes of MIS vs. open SBR with anastomosis were compared before and after propensity score matching (PSM).

**Results:**

Overall, 45 patients with SBR for isolated small bowel pathology were identified, of whom 7 (15.6%) underwent MIS. Of these (3 females, 4 males; median 70, range 29–77 years), six were diagnosed with haemorrhagic small bowel infarction and one with obstructive ileus. Postoperative Comprehensive Complication Index (CCI; median 8.7, range 0-30.8; *p* = 0.04) and time to first flatus (median 2, range 1–4 days; *p* = 0.02) were lower after MIS compared to OS. Postoperative diet progression (median 4.5, range 2–7 days) and length of stay (median 6.5 days, range 4–12 days) did not differ (length of stay: *p* = 0.34, diet progression: *p* = 0.11). There were no anastomotic leaks or revisional surgeries after MIS. The costs for MIS were lower compared to OS (EUR 5,975 vs. EUR 8,925). There were no group differences after PMS.

**Conclusion:**

Our data suggest that laparoscopic small bowel resections with intracorporal anastomosis in an emergency setting are feasible and safe in highly selected cases.

## Introduction

Minimally invasive surgery has become standard practice for many complex abdominal procedure [1], and is also frequently employed in emergency situations (such as appendicitis and cholecystitis, but also more complex pathologies such as oesophageal perforations or sigmoid diverticulitis [2–5]. Laparoscopy has several advantages over the open approach including reduced postoperative pain [6], wound infection rates [6–10] and length of stay [6, 8, 9]. In the long term, a reduction of adhesion-related bowel obstructions[11–13] and incisional hernias [13] are observed.

For instance, in emergency colorectal surgery, the laparoscopic approach has been shown to be associated with lower complication rates and a shorter hospital stay while postoperative morbidity and mortality were comparable to those associated with the open approach [14–16]. A similar trend has been observed in trauma surgery, where laparoscopy has been associated with reduced wound infections, faster recovery and shorter hospital stays, even in complex cases [17]. Comparable benefits have also been reported in the emergency management of groin hernias [18].

The broad spectrum of indications for isolated small bowel resection during emergency surgeries mainly includes gangrene due to incarcerated hernias, acute small bowel obstruction (ASBO), haemorrhagic small bowel infarction or perforated jejunal diverticula [19]. In these situations, the current standard approach remains open surgery (OS). Successful laparoscopic management has been sporadically reported for selected cases, such as primary small bowel volvulus [20], or laparoscopic ileocecal resection with intracorporal anastomosis in a patient with free perforation and faecal peritonitis due to undiagnosed Crohn’s disease [21].

Existing evidence suggests that OS for small bowel resections with primary anastomosis can be considered safe, even in emergency settings, with low reported rates of anastomotic leakage [22, 23]. However, there is little evidence regarding the use, feasibility and safety of laparoscopic SBR with primary intracorporal anastomosis in emergency situations.

Relative contraindications to laparoscopy include prior multiple abdominal surgeries, damage-control scenarios and significant bowel distension. Additional concerns include limited intraoperative visibility, potential hemodynamic compromise due to capnoperitoneum and an elevated risk of inadvertent bowel injury. To date, no formal guidelines recommend the laparoscopic approach, and its use is generally restricted to experienced surgeons [24].

Recent research has primarily focused on the management of ASBO. Despite the increased risk of iatrogenic bowel injury, laparoscopy is increasingly used in selected patients, particularly in those with limited adhesions [25–27, 27]. In cases of ASBO requiring SBR, laparoscopic intervention has been associated with fewer postoperative complications and shorter hospital stay [28]. A more extensive retrospective analysis confirmed overall benefits of MIS over OS in SBR regarding morbidity and mortality [29]. However, this analysis did not specify the indications for SBR, nor did it focus on emergency cases.

## Hypothesis and research question

In this retrospective study, we compared outcomes for patients undergoing MIS vs. OS emergency SBR with primary anastomosis due to isolated small bowel pathology. The study evaluates several outcome parameters such as the Comprehensive Complication Index (CCI), recovery parameters, complication rates, and costs.

## Methods

All consecutive cases involving SBR with intracorporal anastomosis in an emergency setting at our tertiary referral university centre (Department of Visceral Surgery of the University Hospital of Wuerzburg) during 01/2020–04/2025 were collected. A comparative analysis was then conducted between patients who underwent minimally invasive surgery (MIS) versus open surgery (OS). The primary endpoint was the Comprehensive Complication Index (CCI). Secondary endpoints included length of hospital stay, time to first postoperative flatus, diet progression to light regular diet, as well as the rate of anastomotic leakage, revision surgeries and wound complications. To reduce selection bias and improve comparability between the MIS and OS groups a Propensity score matching (PSM) was performed, including the key variables ASA classification, Charlson Comorbidity Index, presence of ileus, and prior abdominal surgery.

The choice of surgical approach (MIS versus OS) was made by the attending on-call surgeon on an individual basis and was not standardized. The decision depended on the presumed technical feasibility of a minimally invasive approach and the surgeon’s experience and expertise with laparoscopic bowel resection. Consequently, treatment allocation was not randomized, and the present analysis reflects real-world clinical practice. All eligible emergency SBR cases during the study period were initially identified and subsequently screened according to the inclusion and exclusion criteria listed below.

### Inclusion and exclusion criteria

Patients were eligible for inclusion if they underwent emergency SBR with primary anastomosis during on-call hours (i.e., night shifts or weekends). The study focused on cases with isolated small bowel pathology.

Eligible indications included:

- ASBO.
- Incarcerated hernia with ischemia or gangrene of the small intestine (including simultaneous hernia repair).
- Jejunal diverticulum perforation.
- Bowel infarction due to venous thrombosis.

Exclusion criteria encompassed:

- Age < 18 years.
- Tumor-associated intestinal perforations.
- Abdominal compartment syndrome.
- Polytrauma or penetrating abdominal trauma.
- Early postoperative complications requiring re-exploration.
- Mesenteric ischemia.
- Resections extending beyond a small bowel segment.
- Extensive intra-abdominal adhesions (“frozen abdomen”).
- Acute severe systemic illness (e.g. sepsis, graft-versus-host disease, myocarditis, pancreatitis, common variable immunodeficiency).
- Cases requiring conversion from MIS to OS.

### Procedure

All MIS procedures were performed by experienced laparoscopic surgeons in the supine position. After the damaged segment of the small intestine was identified, a tubular resection was performed using ultrasonic scissors. The small intestine was then transected transversely using an endoscopic linear stapler device, with both ends subsequently closed. A slight extension of a 12 mm trocar incision site was made for specimen retrieval. For reconstruction, a linear stapler assisted latero-lateral anastomosis was performed. The enterotomy was subsequently closed with a running suture.

During the enterotomy for the side-to-side small bowel anastomosis, special attention was paid to meticulously avoid leaking of small bowel contents into the abdominal cavity. This was achieved by an immediate use of a suction device by the assistant during the opening process of the small bowel.

When contaminated fluids in the abdominal cavity caused by bacterial translocation due to severe damage of the small intestine were present, extensive laparoscopic lavage was performed, especially in case of mesh placement for incarcerated inguinal hernia [30].

Open procedures were carried out by a specialist registrar or the attending surgeon on call.

Analogous to MIS, the damaged segment was identified and tubularly resected with adequate proximal and distal margins. The transection was performed using a linear stapler. Reconstruction was achieved by creating a side-to-side anastomosis using the same stapling device. The enterotomy was also closed with a running suture.

## Results

In total, 361 patients underwent emergency SBR, of whom 45 (12.5% of the total) were eligible for inclusion based on the inclusion and exclusion criteria. Of these 45 patients, seven (15.6% of the selected cases, 1.9% of the total) underwent MIS.

### Patient characteristics

Patient characteristics are summarized in Table 1. Age (*p* = 0.28) and BMI (*p* = 0.24) were comparable between groups. Gender distribution did not differ significantly (MIS: 3 females/4 males, OS: 18 females/20 males; X² = 0.05, df = 1, *p* = 0.83). Comorbidity burden, assessed via the Charlson Comorbidity Index, showed a trend toward higher scores in the OS group (*p* = 0.07), but did not reach statistical significance. ASA classification was similar in both groups (*p* = 1). Notably, prior abdominal surgery was significantly more frequent in the OS group compared to the MIS group (*p* = 0.05). The diagnosis ileus at the time of emergency surgery was equally often established in both groups (*p* = 0.76).

**Table 1:** Patient baseline characteristics

	OS (*n* = 38) median (range)	MIS (*n* = 7) median (range)	Test statistic	*P*-value
Age	70.5 (27–93)	70 (29–77)	U = 98	*p* = 0.28
BMI	24.8 (17.8–48.6)	28.1 (22.8–31.7)	U = 129.5	*p* = 0.24
Charlson Comorbidity Index	4 (0–11)	3 (0–6)	t = -1.86, df = 43	*p* = 0.07
ASA	3 (1–4)	3 (2–4)	U = 133.5	*p* = 1
Prior abdominal surgery	*n* = 30	*n* = 3	X² = 3.94, df = 1	*p* = 0.05 *
Ileus	*n* = 24	*n* = 4	X² = 0.09, df = 1	*p* = 0.76

The indications for SBR were, in the MIS group, four cases of small bowel haemorrhage gangrene due to incarcerated hernia, two cases of infarction due to isolated mesenteric venous thrombosis, and one case of ASBO; in the OS group, there were 17 cases of ASBO, 10 cases of perforated jejunal diverticulum, six cases of small bowel haemorrhage gangrene due to incarcerated hernia, three cases of infarction due to isolated mesenteric venous thrombosis, one case of volvulus, and one case of unclear small bowel obstruction without evidence of malignancy.

### MIS case reports

#### Case 1

A 72-year-old man with segmental haemorrhagic small bowel ischemia caused by partial portal vein thrombosis of unknown origin. His history entailed coronary heart disease (CHD), condition after deep vein thrombosis (DVT) and recurrent syncope (Charlson Comorbidity Index 5). The patient underwent MIS resection of 38.5 cm Jejunum and a latero-lateral jejuno-jejunostomy. During his postoperative course he experienced a bowel paralysis and trocar side infection which both were treated conservatively (Clavien-Dindo Index 21.8).

#### Case 2

**Fig. 1:**
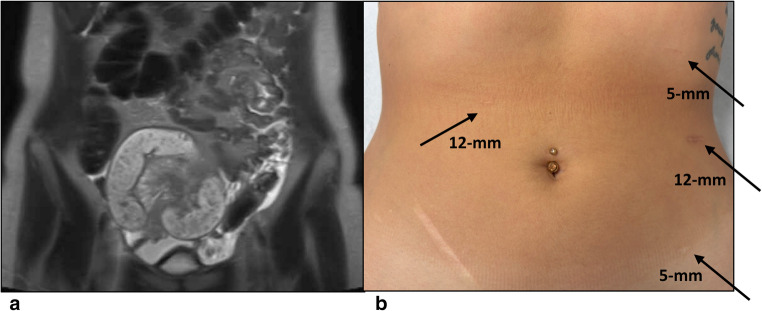
**a **– MRI with ASBO after open appendectomy (case 2). **b **– Postoperative cosmetic result with minimal scarring

A 29-year-old healthy (Charlson Comorbidity Index 0) female patient with haemorrhagic small bowel infarction due to strangulation obstruction of the terminal ileum after open appendectomy (see Fig. 1). After resection of 31 cm ileum a latero-lateral ileo-ileostomy was performed. The postoperative course was uneventful (Clavien-Dindo Index 0).

#### Case 3

A 74-year-old female patient with haemorrhagic small bowel gangrene due to an incarcerated femoral hernia. Her medical history entailed myocardial infarction and CHD (Charlson Comorbidity Index 4). After resection of 8.5 cm jejunum a latero-lateral jejuno-jejunostomy as well as a left TAPP for hernia repair was performed. During the postoperative course a hypokalemia occurred (Clavien-Dindo Index 8.7).

#### Case 4

A 70-year-old healthy female patient (Charlson-Comorbidity Index 3) with focal small bowel perforation due to incarcerated inguinal hernia. After resection of a 9 cm jejunal segment and a right-sided TAPP was performed. Postoperatively, the patient developed a right flank phlegmon and trocar site infection, which were managed by antibiotic therapy. Additionally, a left-sided DVT with bilateral pulmonary embolism (PE), requiring initiation of full anticoagulation (Clavien-Dindo Index 30.8) occurred.

#### Case 5

A 54-year-old male patient with partial haemorrhagic small bowel infarction due to thrombosis of the superior mesenteric vein. His medical history entailed arterial hypertension (Charlson-Comorbidity Index 1). Surgical treatment included resection of a 59 cm jejunal segment with consecutive latero-lateral jejunojejunostomy. There were no postoperative events (Clavien-Dindo Index 0). Further evaluation could not determine the etiology of the thrombotic event, and the thrombosis was ultimately classified as idiopathic.

#### Case 6

A 77-year-old female patient (Charlson Comorbidity Index 6) with small bowel gangrene caused by an incarcerated trocar site hernia after laparoscopic sigmoid colon cancer resection. The surgical intervention involved resection of an 18 cm segment of the jejunum. The early postoperative course was complicated by the occurrence of atrial fibrillation, full anticoagulation was initiated (Clavien-Dindo Index 20.9).

#### Case 7

A 43-year-old healthy (Charlson Comorbidity Index 0) male patient with segmental small bowel gangrene due to an incarcerated inguinal hernia. Surgical management included minimally invasive ileal segment resection (23.5 cm) followed by (open) hernia repair. The postoperative course was uneventful (Clavien-Dindo Index 0).

Concomitant surgical procedures beside the SBR in MIS group entailed simultaneous hernia repairs. In detail, two laparoscopic transabdominal preperitoneal (TAPP) and one open (Lichtenstein) repairs for inguinal hernias and the repair of a trocar hernia.

Concomitant surgical procedures beside the SBR in OS group entailed the simultaneous repair of two umbilical, two inguinal, one incisional and one epigastric hernia repairs. Furthermore, re-positioning of both a continuous ambulatory peritoneal dialysis (CAPD) catheter and a ventriculoperitoneal (VP) shunt was performed. Further, a bowel re-resection with creation of a new latero-lateral anastomosis (after previous bariatric surgery), as well as excision of a cutaneous tumor were conducted (Table 2). 

**Table 2:** Summary of MIS cases

Case	Age, sex	Indication	CCI	Proc.	Resec. length	Additional procedures	Postoperative complications
1	72 Male	Haemorrhagic small bowel ischemia due to partial portal vein thrombosis	5	Jejunal resection	38.5 cm	—	Postoperative bowel paralysis, trocar site infection
2	29 Female	Strangulation obstruction of terminal ileum (post-appendectomy)	0	Ileal resection	31 cm	—	None
3	74 Female	Haemorrhagic small bowel gangrene due to incarcerated femoral hernia	4	Jejunal resection	8.5 cm	TAPP (left)	Hypokalemia
4	70 Female	Small bowel perforation due to incarcerated inguinal hernia	3	Jejunal resection	9 cm	TAPP (right)	Trocar site infection, phlegmon; DVT with bilateral PE
5	54 Male	Haemorrhagic small bowel infarction due to superior mesenteric vein thrombosis	1	Jejunal resection	59 cm	—	None
6	77 Female	Small bowel gangrene due to incarcerated trocar site hernia (post colorectal surgery)	6	Jejunal resection	18 cm	—	Postoperative atrial fibrillation
7	43 Male	Small bowel gangrene due to incarcerated inguinal hernia	0	Ileal resection	23.5 cm	Open hernia repair	None

Abbreviations: *CCI* Charlson-Comorbidity Index, *DVT* Deep Vein Thrombosis, *Resec.length* Resection length

### Operative outcome

In the MIS group, two patients (28.6%) underwent ileoileostomy and five patients (71.4%) jejunojejunostomy. In the OS group, 16 patients (42.1%) had ileoileostomy and 21 patients (55.3%) jejunojejunostomy, with one case missing detailed description of resection location (2.6%). Overall, 18 patients received ileoileostomy and 26 underwent jejunojejunostomy. There was no significant difference in the distribution of anastomosis localization between MIS and OS groups (χ² = 0.45, df = 1, *p* = 0.50). Resection length (*p* = 0.63) and operation time (*p* = 0.16) were comparable between MIS and OS groups (Table 3).

**Table 3:** Operative outcome

	OS (*n* = 38) median (range)	MIS (*n* = 7) median (range)	Test statistic	*P*-value
Resection length(in cm)	17.25 (3.5–120)	23.5 (8.5–59)	U = 149	*p* = 0.63
Operation time (in minutes)	78.5 (33–304)	101 (70–174)	U = 178	*p* = 0.16

### Primary endpoint

**Fig. 2:**
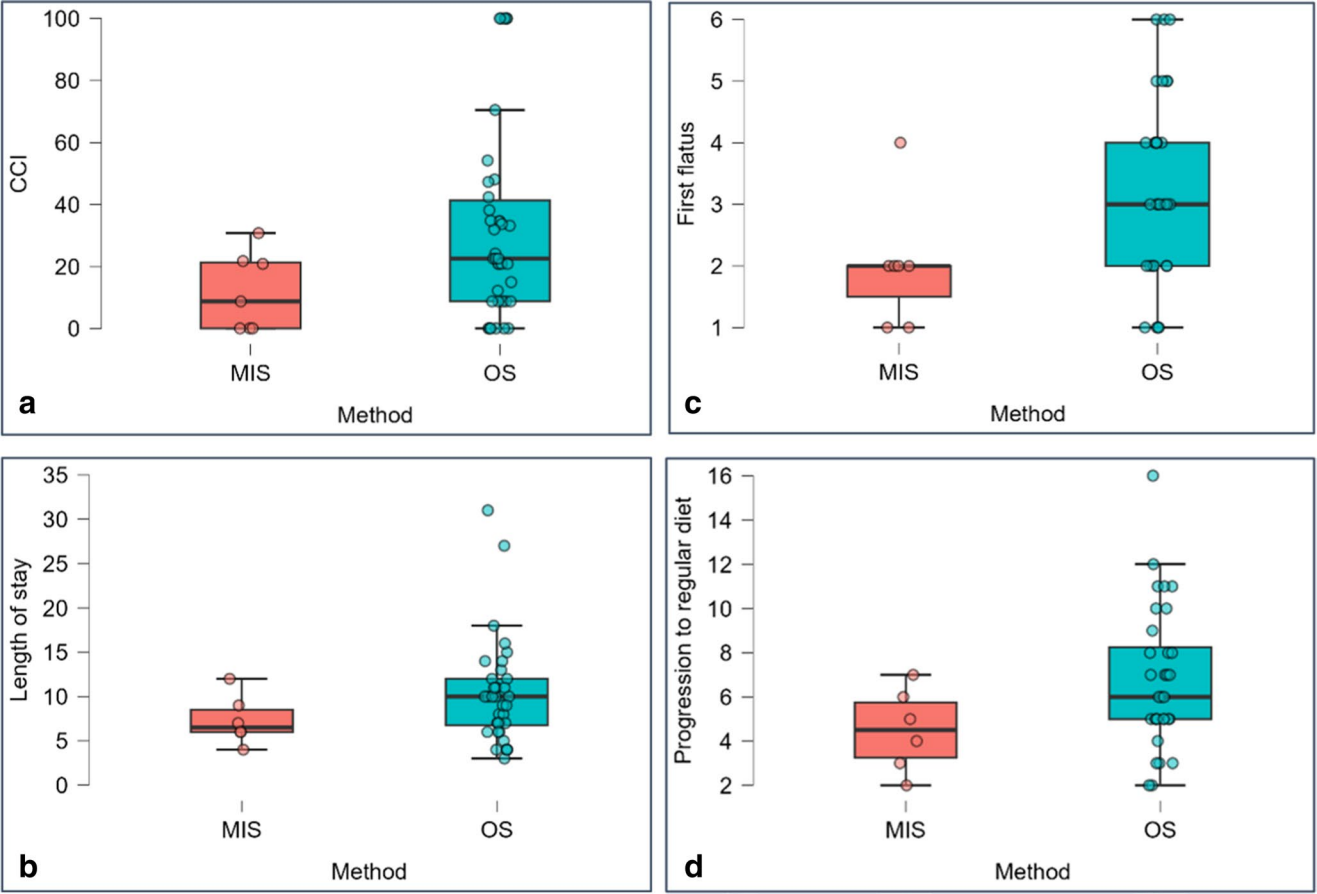
**a** – Comparison of CCI (p = 0.04 *). **b** – Postoperative length of stay (p = 0.34). **c** – First flatus (*p* = 0.02 *). **d** – Progression to regular diet (*p* = 0.11)

Postoperative complications were evaluated using the Comprehensive Complication Index (CCI). The median CCI was 8.7 (range 0–30.8) in the MIS group and 22.6 (range 0–100) in the OS group. In multivariable linear regression analysis, the surgical approach (MIS vs. OS) was an independent predictor of postoperative complications (β = − 23.6, *p* = 0.04), with MIS predicting lower CCI values. Additionally, preoperative ileus (β = 20.3, *p* = 0.02) showed a significant positive association with CCI, while mesh implantation demonstrated a trend towards higher complication scores (β = 16.6, *p* = 0.09) (Fig. 2).

### Secondary endpoints

#### Length of stay

MIS was not associated with a reduction in length of stay (β = − 3.41 days, *p* = 0.34). Median length of stay was 6.5 days (range 4–12) in the MIS and 10 days (range 3–50) in the OS group. Additionally, preoperative ileus (β = 4.99, *p* = 0.05) showed a slightly significant positive interaction in prolonging length of stay.

There were no differences in length of stay between Intensive Care Unit (ICU: median 0, range 0–8, W = 158.5, *p* = 0.39), Intermediate Care Unit (IMC: median 0, range 0–9, W = 138.5, *p* = 0.85), and Normal Ward (Median 8, range 0–40, W = 133.5, *p* = 1.00).

#### First flatus

Patients undergoing MIS had a median time to first flatus of 2 days (range 1–4), compared to 3 days (range 1–6) in the OS group. Multivariable regression analysis revealed MIS being significantly associated with a shorter time to first flatus (β = –1.35, p = 0.02), while the occurrence of ileus was linked to a delayed first flatus (β = 1.12, p = 0.02). 

#### Progression to regular diet

Progression to regular diet was comparable between MIS (median 4.5 days, range 2-7) and OS (median 6 days, range 2–16) group. In a generalized linear model, MIS was not associated with faster diet progression (β = –2.16 days, *p* = 0.11). Preoperative ileus was significantly associated with delayed diet progression, increasing the time by approx. 2 days on average (β = 1.97, *p* = 0.048) (Table 4). 

**Table 4:** Primary and secondary endpoints

	OS (*n* = 38) median (range)	MIS (*n* = 7) median (range)	*P*-value
CCI-Score	22.6 (0-100)	8.7 (0-30.8)	*p* = 0.04 *
Length of stay (in days)	10 (3–50)	6.5 (4–12)	*p* = 0.34
First flatus (in days)	3 (1–6)	2 (1–4)	*p* = 0.02 *
Diet progression (in days)	6 (2–16)	4.5 (2–7)	*p* = 0.11

In the MIS group, all 7 procedures (100%) were performed by consultants. Conversely, in the OS group, 12 out of 38 procedures (31.6%) were primarily performed by residents and 26 (68.4%) by consultants. A statistically significant association was identified between surgical method and surgeon level of expertise (χ² = 6.82, df = 1, *p* = 0.009 **). 

#### Surgical site complications

Notably, two patients (28.6%) in the MIS group developed phlegmonous trocar site infections, which were successfully treated with antibiotics. In the OS group, superficial wound infections (SSI Grade 1, n = 6, 15.8%) and deeper surgical site complications (SSI Grade 2-3, n = 4, 10.5%) were observed. In the OS group, the localized superficial wound infections required local treatment (including open wound treatment and wound irrigation), while in the MIS group, superficial phlegmonous infections (originating from trocar site) were treated with systemic antibiotics. 

There were no revisional surgeries after MIS. After OS four patients required revisional surgery including three cases of deeper wound complications (infection on a mesh implant site, burst abdomen, intraabdominal abscess, χ² = 0.59, df = 1, *p* = 0.44) and one case of anastomotic leakage (χ² = 0.19, df = 1, *p* = 0.66). In the latter case, best supportive care was initiated due advanced age and patient’s will (Table 5).

**Table 5:** Surgical site infection management

		MIS (*n* = 7) *n* (%)	OS (*n* = 38) *n* (%)	Test statistic	*P*-value
SSI	Grade 0	5 (71.4)	28 (73.7)	---	*p* = 0.59
	Grade 1	2 (28.6)	6 (15.8)		
	Grade 2	0 (0)	1 (2.6)		
	Grade 3	0 (0)	3 (7.9)		
Local Therapy		0 (0)	8 (21.1)	OR = 0, 95%CI [0-3.28]	*p* = 0.32
Systemic Therapy		2 (28.6)	2 (5.3)	OR = 6.71, 95%CI [0.40-112.94]	*p* = 0.11
Surgical Revision / Intervention		0 (0)	3 (7.9)	OR = 0, 95%CI [0-14.16]	*p* = 1

#### Cost calculation MIS vs. OS

**Fig. 3:**
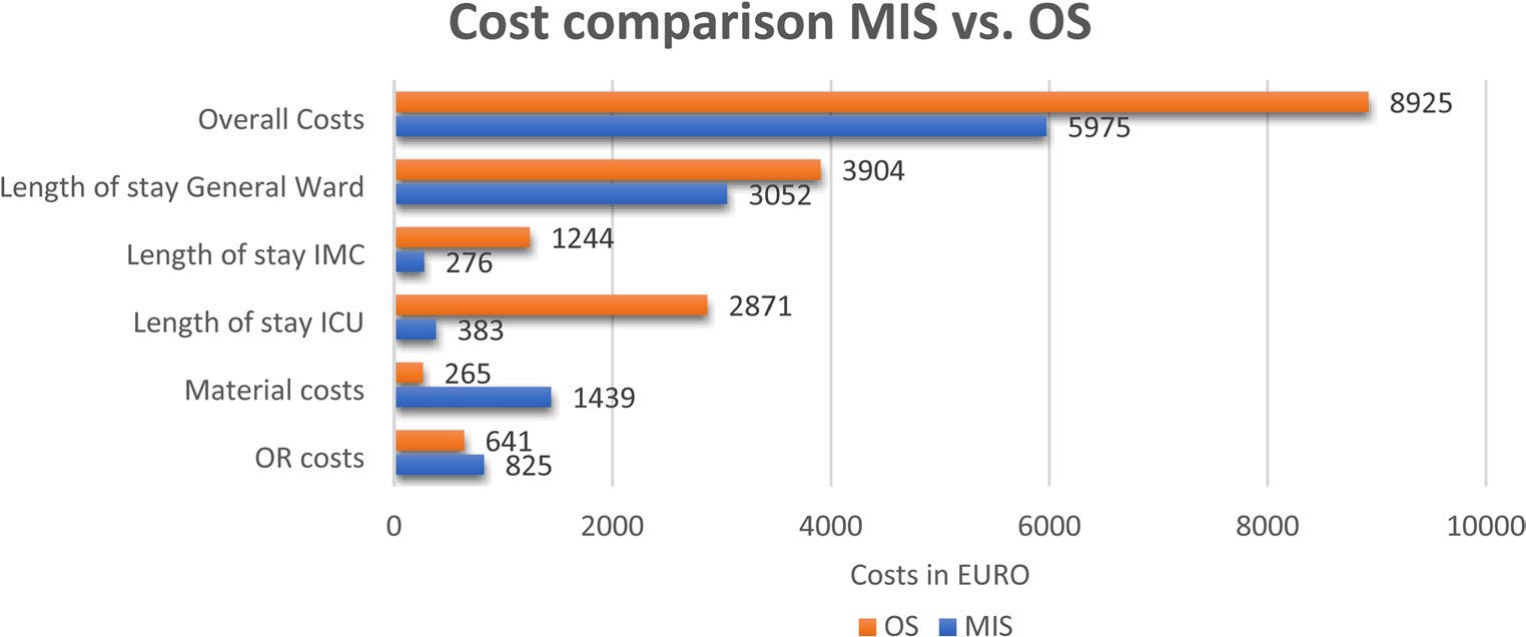
Cost effectiveness (in EURO)

To assess the cost-effectiveness of MIS vs. OS [31, 32], we compared the costs for the operation itself (including materials and costs for operating the OR) and the hospital stay, using average values. Intraoperative personnel costs were not considered as the same staffing levels were assumed.The intraoperative material costs for MIS were clearly higher than for OS (EUR 1439 vs. EUR 265), which was driven by expenses for the stapler (MIS: Endoscopic Linear Stapler with three reload units; OS: Linear Stapler with one reload unit). Additionally, OR costs were higher for MIS than for OS due to longer operating times (EUR 825 vs. EUR 641). To calculate the costs associated with the hospital stay, the mean length of stay for each unit (Intensive Care Unit (ICU) vs. Intermediate Care Unit (IMC) vs. Normal Ward) was multiplied by the overall costs of providing and supplying a hospital bed in the according unit (including nursing staff, water, heating, electricity, etc.). Hospital stay costs were lower for MIS than for OS (EUR 3711 vs. EUR 8019) due to an overall shorter and lower length of stay in the ICU or IMC unit. On average, costs were lower for MIS patients than for OS patients (EUR 5975 vs. EUR 8925), representing a cost saving of approximately one third per patient (EUR 2950) (Table 6; Fig. 3).

**Table 6:** Cost effectiveness (in EURO, values rounded to whole numbers)

		Factor	MIS	Factor	OS
Operating room costs	EUR 8 per minute	101 min	825	78.5 min	641
Material costs			1439		265
Length of stay in Intensive-Care-Unit	EUR 1914 per day	0.2 days	383	1.5 days	2871
Length of stay in Intermediate-Care-Unit	EUR 1382 per day	0.2 days	276	0.9 days	1244
Length of stay in Normal Ward	EUR 449 per day	6.8 days	3052	8.7 days	3904
Overall costs			5975€		8925€

### Propensity score matching

Propensity score matching (PSM) was performed to reduce selection bias and improve comparability between the MIS and OS groups. Propensity scores were estimated using a logistic regression model including ASA classification, Charlson Comorbidity Index, presence of ileus, and prior abdominal surgery. Patients with missing values in any of these covariates were excluded from the matching procedure (complete-case analysis).

Several matching specifications were evaluated, including 1:1, 1:2, and 1:3 matching ratios. Nearest-neighbor matching without replacement was applied using a caliper width of 0.4 standard deviations of the logit of the propensity score. Although 1:2 and 1:3 matching increased the number of matched controls (8 and 9 controls, respectively), this gain in sample size was accompanied by less favorable balance diagnostics Therefore, the 1:1 matching approach yielded the lowest overall residual imbalance and was selected for the primary analysis.

Before matching, substantial baseline imbalances were present between MIS and OS patients, particularly with respect to comorbidity burden (Charlson Comorbidity Index; |SMD| = -0.70) and prior abdominal surgery (|SMD| = 0.73). Primary 1:1 matching identified six matched pairs from seven MIS patients and 38 OS patients, resulting in a final matched cohort of 12 patients. One MIS patient and 32 OS patients remained unmatched.

**Fig. 4:**
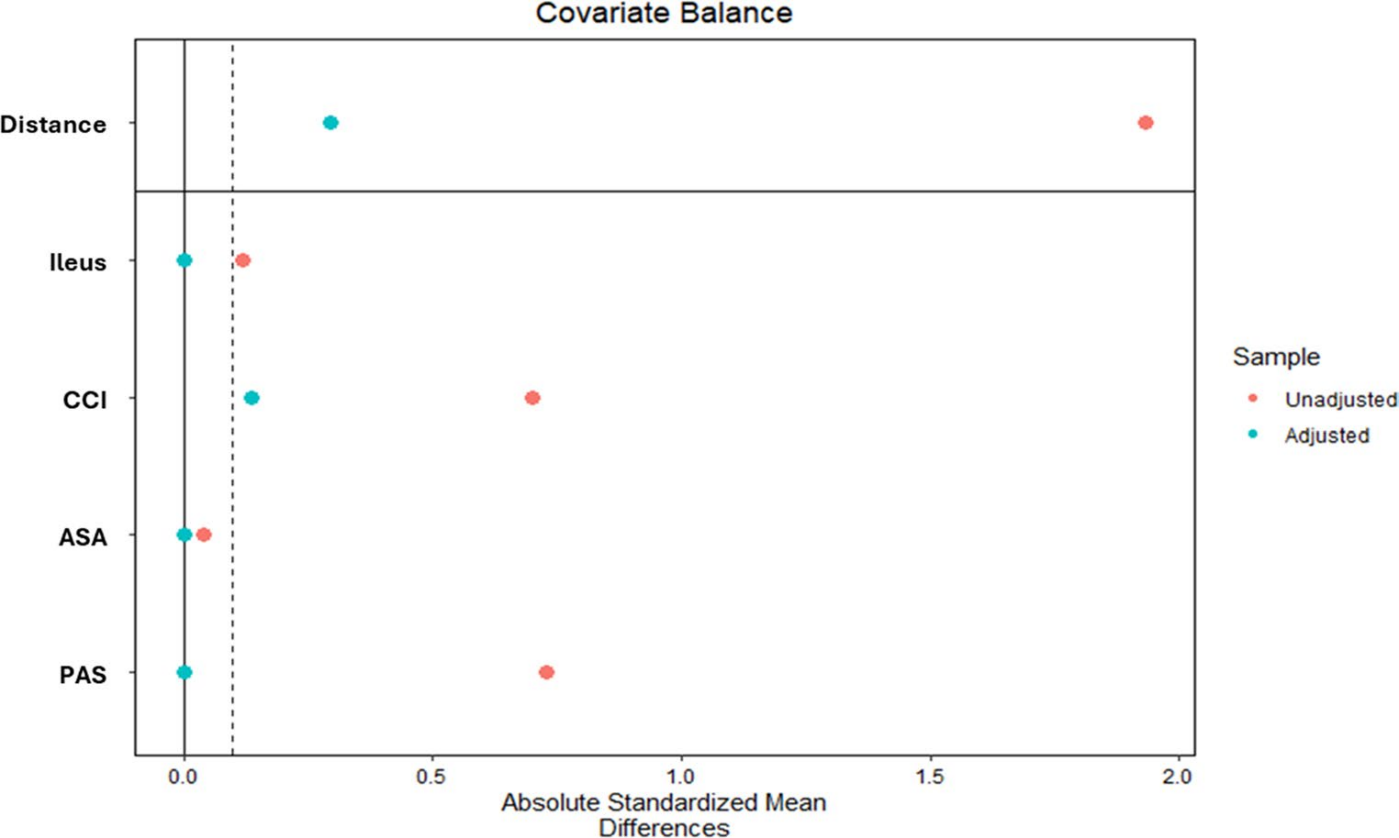
Standardized mean differences before and after 1:1 propensity score matching. Abbreviations: ASA, American association of anesthesiologists score; CCI, Charlson comorbidity index; PAS, Previous abdominal surgery. This Love plot illustrates the distribution of standardized mean differences (SMD) for all baseline covariates before and after 1:1 nearest-neighbor propensity score matching. After matching, SMD values were markedly reduced for all covariates achieving acceptable balance, indicating improved comparability between MIS and OS

In the final matched cohort, exact balance was obtained for ileus, ASA classification, and prior abdominal surgery (all |SMD| = 0.00), while a minor residual imbalance remained for the Charlson Comorbidity Index (|SMD| = -0.14). Consequently, the substantial pre-matching differences in comorbidity burden and prior abdominal surgery were markedly reduced. The caliper width of 0.4 provided a favorable balance between covariate balance and sample retention, allowing 6 of 7 MIS patients (85.7%) to be successfully matched while maintaining sufficient post-matching balance across all prespecified covariates (Fig. 4). 

After PSM, a sufficient balance between the MIS and OS group was achieved, allowing for an additional comparative analysis. No statistically significant differences were observed between the groups with respect to the Charlson Comorbidity Index (CCI) score (χ² = 9.33, df = 7, *p* = 0.23), length of hospital stay (χ² = 7.33, df = 8, *p* = 0.50), time to first flatus (χ² = 5.20, df = 4, *p* = 0.27), and time to diet progression (χ² = 6.67, df = 7, *p* = 0.46).

These findings demonstrate that, within a well-matched cohort, the MIS approach is not associated with inferior outcomes compared to OS across key perioperative recovery parameters, thereby supporting non-inferiority of MIS in this context.

## Discussion

Our data demonstrate that laparoscopic SBR with intracorporal anastomosis in the emergency and acute care setting is feasible and safe in selected cases with appropriate MIS expertise.

In our cohort, MIS was associated with a lower overall postoperative morbidity, as measured by the Comprehensive Complication Index (CCI) and earlier recovery of bowel function compared to OS, while length of hospital stay was comparable between groups. The latter may in part be explained by the fact that no standardised discharge criteria were established for these emergency surgeries during the observation period, where hospital stay could substantially be shortened for MIS (as suggested in other settings) [33]. Preoperative ileus represents an important confounding factor, as it was associated with postoperative recovery and complications in our cohort. Although this was addressed by propensity score matching, residual confounding inherent to the retrospective study design cannot be completely excluded. Taken together, and given the non-randomized / non-standardized treatment allocation, these findings should be interpreted as associative rather than causal.

Notably, we observed an unexpectedly high rate of phlegmonous infections near the trocar sites (even distant to the extraction site) after MIS, possibly, as a result of a site contamination during the retrieval of the resected and damaged small intestine or by contaminated ascites due to the pressure of the capnoperitoneum [34, 35]. However, in MIS all of these were successfully treated with antibiotic treatment. Risk factors associated with the development of SSIs include suboptimal or inadequate antibiotic prophylaxis, female sex, elevated BMI, and perioperative hyperglycemia [36]. Previous studies have shown a high rate of local wound complications even in elective settings when contaminated bowel segments/staplers were used without a protective sleeve to retrieve the specimen [37]. Thus, also in MIS, wound protection is a key measure to prevent SSIs.

Potential long-term benefits of MIS that have been reported for minimally invasive approaches in other settings, but were not assessed in the present study due to the short-follow up period and retrospective study design, may include reduced rates of adhesions and incisional hernias over time, thereby potentially lowering long-term healthcare costs and reducing the burden on hospital resources [38, 39]. 

A limitation of this study is the unequal distribution of surgeon experience between OS and MIS group. All MIS procedures were performed by consultants, whereas a substantial proportion of OS was performed by residents under supervision. This may have influenced perioperative outcomes independently of the surgical approach and therefore represents a potential source of confounding. However, a notable disadvantage of applying MIS in these situations is the potential reduction in training opportunities, as these surgeries represent appropriate supervised training procedures, performed by residents under the guidance of a consultant. The increased adoption of MIS may therefore shift these training interventions toward a specialization, potentially endangering a valuable part of surgical education [40]. 

Notably, MIS is associated with considerably higher material costs [41–43], which can be refinanced when defined discharge criteria lead to a shorter hospital stay or to avoidance of ICU/IMC unit observation [44].The retrospective design and the non-randomized allocation of the surgical approach introduce an inherent risk of selection bias. The decision to perform MIS or OS was made by the attending surgeon based on clinical judgement, technical feasibility, and individual expertise. Therefore, residual confounding and differences in baseline characteristics between groups cannot be fully excluded despite the applied eligibility criteria.

To address the inherent baseline imbalances between the MIS and OS groups, propensity score matching was applied. The matching process reduced pre-existing differences in key confounders, particularly preoperative ileus, comorbidity burden and prior abdominal surgery. The final 1:1 matched cohort achieved sufficient covariate balance across most prespecified variables, suggesting that the observed postoperative differences are less likely to be driven by measurable baseline characteristics. Nevertheless, it is important to acknowledge that the relatively small number of successfully matched patients inevitably limits statistical power. A further limitation of the propensity score analysis is that the underlying surgical indication was not included in the matching procedure. Although indication is a clinically relevant determinant of treatment allocation and postoperative outcomes, the heterogeneity of indications combined with the very small number of patients in the MIS group precluded reliable matching without substantial loss of the study population. Consequently, confounding related to indication cannot be excluded and should be considered when interpreting the results.

 We further admit that the presented case series represents only a small portion of all emergency SBR. Beside the availability of a fully trained laparoscopic surgeon there are many other reasons why the open over the minimally-invasive approach was chosen in these situations. Time constrains in cases of several outstanding emergencies, extensive previous open surgery and/or extensive small bowel obstruction. Therefore, the results of this paper should be interpreted as hypothesis-generating and supportive of the main findings rather than confirmatory evidence of causality.

## Conclusion

Laparoscopic SBR with intracorporal anastomosis in an emergency setting appears feasible and safe in highly selected cases when appropriate expertise is available. Attention needs to be paid to trocar site infections. While MIS was associated with favorable short-term postoperative outcomes in our cohort, the retrospective design and potential selection bias preclude conclusions regarding superiority over OS. Therefore, the role of laparoscopy in emergency SBR remains to be defined. Prospective studies are required to identify those patient subgroups that are most likely to benefit from a minimally invasive approach and to develop strategies that minimize intraoperative and postoperative risks.

## Data Availability

No datasets were generated or analysed during the current study.

## Declarations

This research study was conducted retrospectively from data obtained for clinical purposes. It was conducted in accordance with the ethical standards of the institutional and national research committee and with the 1964 Helsinki Declaration and its later amendments or comparable ethical standards. Ethical approval was given by the Ethics Committee of the University of Würzburg (2026-170-dvhd).
